# Study on Simulation and Experiment of Cu, C-Doped Ag/Ni Contact Materials

**DOI:** 10.3390/ma15114019

**Published:** 2022-06-06

**Authors:** Ying Zhang, Jingqin Wang, Yancai Zhu, Defeng Cui, Ningyi Lu

**Affiliations:** 1 State Key Laboratory of Reliability and Intelligence of Electrical Equipment, Hebei University of Technology, Tianjin 300130, China; 13512474229@163.com (Y.Z.); zhuyc@hebut.edu.cn (Y.Z.); 2 Guilin Electrical Equipment Scientific Research Institute Co., Ltd., Guilin 541000, China; cuidf10@126.com; 3 Xiamen Hongfa Electroacoustic Co., Ltd., Xiamen 361000, China; lu_ny@hongfa.cn

**Keywords:** Ag/Ni contact material, density functional theory, doping, interfacial bonding strength

## Abstract

Ag/Ni contact material with greenery and good performance is a cadmium-free silver-based contact material that has been vigorously developed in recent years. However, Ag/Ni contact material has poor welding resistance. Based on the first principles of density functional theory, the interface model of Cu, C-doped Ag/Ni was established. The work of separation and interfacial energy of interface models showed that doping can improve the interfacial bonding strength and interfacial stability, with C-doped Ag/Ni having the strongest stability and interfacial bonding strength. It can be seen from the population and density of state that covalent bonds exist between Ag and Ni atoms of the Ag/Ni phase interface at the electronic structure level. Finally, the doped Ag/Ni contact material was prepared by the powder metallurgy method. Through the arc energy and welding force in the electrical contact experiment, it was obtained that the welding resistance of C-doped Ag/Ni was better than Cu-doped Ag/Ni contact material, which verified the correctness of the simulation results. Overall, the present study provides a theoretical method for the screening of doping elements to improve the performance of Ag/Ni contact material.

## 1. Introduction

As the core component of the low-voltage switch, the electrical contact plays the role of opening and closing electric circuits, and electrical contact materials affect the reliability and service life [1,2]. Therefore, it is of great significance to study electrical contact materials. Ag/CdO as a “universal contact material” is widely utilized in various situations because of its excellent performance. However, CdO decomposes toxic cadmium vapor at high temperatures, which poses a great risk to human health and environmental safety. The internationally accepted environmental protection directive is the RoHS issued by the European Union. It requires that six hazardous substances, including cadmium (Cd), should be banned from manufacture and import of electrical and electronic products from July 2006. Although, the annex to the RoHS Directive exempts the use of Cd and its compounds in electrical contacts. However, in 2019, the EU further revised the RoHS Directive, stipulating that the use of Cd and its compounds in electrical contacts can be exempted until 21 July 2021 for most applications, and a small number of special applications can be exempted until at the latest 21 July 2024 [3,4]. Therefore, the research for environmentally friendly electrical contact materials to replace Ag/CdO has attracted extensive attention.

An environmentally friendly Ag/Ni contact material with good electrical and thermal conductivity and excellent processability has been vigorously developed in recent years. However, Ag/Ni contact material has poor welding resistance, which greatly limits the application of Ag/Ni contact materials [5,6,7]. Therefore, improving the welding resistance of Ag/Ni contact materials is an urgent problem.

Studies have shown that doping can effectively improve the welding resistance of Ag/Ni contact materials. There has been much attention on doping to improve the performance of Ag/Ni. The authors of [8,9,10] studied the effects of different carbon allotropes (carbon nanotube, nano graphene sheet, and graphite) on the microstructure and properties of Ag/Ni contact through experiments. The results showed that all carbon allotropes can improve the welding resistance. Wang et al. [11] conducted experimental research on the properties of W- and WO_3_-doped Ag/Ni contact material. Han et al. [12] found that TiB_2_ doping can effectively improve the arc erosion resistance of Ag/Ni contact materials. Yang et al. [13] carried out the research on the Ag/Ni contact material with doping high melting point additives. You et al. [14] experimentally investigated that the addition of SnO_2_ can significantly improve the properties of the Ag/Ni material. The current research on the doping of additives to improve the performance of Ag/Ni contact materials almost adopts experimental methods. There has been a lack of theoretical basis, and the blind selection of additives is bound to cause various wastes, resulting in large investment and low efficiency. Therefore, it is urgent to find a simulation method for the optimal composition selection of Ag/Ni contact materials. Among the many simulation research methods for the prediction of material properties and the exploration of experimental mechanisms, the first-principles method is increasingly favored by researchers because it does not rely on any empirical parameters and can ensure the credibility of computational results.

This paper proposed the first-principles method to study Ag/Ni contact material. The reason why the Ag/Ni interface model was established for calculation is that Ni and Ag in Ag/Ni alloys are almost insoluble in the solid state and interfacial bonding strength plays an extremely important role in the properties of materials in the application. Studies in [15] have shown that contact layers make it possible to avoid a rise in electrical resistance due to an increase in the number of elements in the micro-converter. Studies in [16] have shown that an arc has region and component preference under the influence of the erosion. This is because the interfacial bonding strength was weakly and easily enriched with impurity atoms, so the arc acted preferentially at the interface. The authors of [6] showed that the total electronic charge in the binary pseudo-alloy Ag/Ni was redistributed only at a small area of the alloy interface, leading to a change in electronic behavior. The authors of [17] showed that the interface was the key to the quality of the composite and directly affected the performance. Therefore, interface models analyze the interfacial structure of Ag/Ni materials from a microscopic atomic perspective, which is of great significance to welding resistance of Ag/Ni contact materials.

Since Cu is beneficial to improve the wettability between liquid silver and the second phase to reduce the tendency of fusion welding, C can prevent fusion welding. Therefore, Cu and C were selected to dope Ag/Ni contact materials. In this paper, the first-principles method based on density functional theory was proposed to simulate the interface model of Cu- and C-doped Ag/Ni. According to the results, the microscopic mechanism of contact materials doped with different elements, Cu and C, was analyzed from the perspective of atomic scale, and then the best-doped elements of Ag/Ni contact materials with excellent performance were obtained. The research of doping on the interfacial bonding strength is important for improving the performance of materials. The relevant theoretical calculations play an irreplaceable and important role in revealing the microscopic interaction mechanism between doping and interface, obtaining the optimal strategy of doping elements and avoiding the blind exploration of experiments.

## 2. Models and Calculation Method

### 2.1. Models

**Figure 1:**
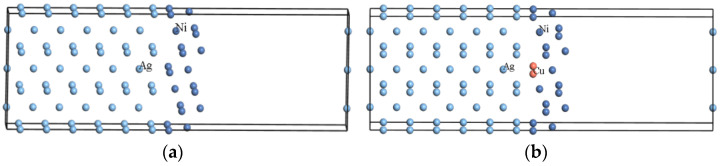
Interface model: (**a**) Ag/Ni, and (**b**) Cu-doped Ag(110)/Ni(211).

Firstly, Ag and Ni unit cells were optimized, respectively, and the stable structure with the lowest total energy was obtained after optimization. Then, the optimized Ag and Ni unit cells were established with the surface models of Ag(110) and Ni(211). Figure 1 shows the Ag/Ni interface model and the Cu-doped Ag/Ni interface model.

Figure 1a shows the Ag/Ni interface model, where the (110) interface is used for Ag and the (211) interface for Ni. The reason for choosing these two interfaces is that the (110) and (211) interfaces of Ag and the (110) and (211) interfaces of Ni can be well-combined in experiments, and then interface models of Ag(110)/Ni(110), Ag(110)/Ni(211), Ag(211)/Ni(110), and Ag(211)/Ni(211) were analyzed by studying the binding energy, charge population, and electronic structure, and the results showed that Ag(110)/Ni(211) was the most stable [18]. Considering the existence of interfacial mismatch in the interface model and the fact that Ag/Ni contact material with a Ni content of approximately 15 wt.% is the most widely utilized in electrical switches, the interface model of Ag(110)/Ni(211) was established. The atomic and mass ratios of the interface models are presented in Table 1. When establishing the interface model, to avoid interactions in the periodic direction, a vacuum layer of 15 Å was added to all the interface models.

An interface model of Ag(110)/Ni(211) was built, and Ni atoms were replaced by a Cu atom and a C atom. Taking Cu doping as an example, the interface model of Cu-doped Ag(110)/Ni(211) is shown in Figure 1b. In this paper, the interface model with a doped element replacing Ni was chosen because Cu and Ni are infinitely miscible, while Cu and Ag are not solidly soluble. C with micro-doping can form a solid solution with Ni and the ratio of Ni atom to C atom diameter is 0.69, while C and Ag are not solid-soluble. Therefore, a Cu atom and a C atom replaced a Ni atom of the Ag/Ni interface model so that Cu, C, and Ni form a solid solution.

### 2.2. Calculation Method

Based on the first-principles method of density functional theory, the CASTEP module of Materials Studio software was used for calculation, and the calculation was carried out in reciprocal space. This simulation is widely utilized in various materials with periodic structures, such as ceramics, semiconductors, and metals [19,20]. The interaction potential between the real ion and the valence electron was described by OTFG ultrasoft pseudopotentials [21]. The exchange correlation potential energy of optimization used the Perdew Burke Ernzerhof (PBE) method under Generalized Gradient Approximation [22]. The BFGS (Broyden Fletcher Goldfarb Shanno) algorithm was used to simulate the Cu, C-doped Ag/Ni interface model.

## 3. Simulation Analysis

First, the interface models were optimized, and then the energy calculation was carried out to analyze the interfacial bonding strength, interfacial stability, and electronic structure of the Cu, C-doped Ag/Ni interface model.

### 3.1. Interfacial Bonding Strength

The work of interfacial separation (*W_sep_*) can be defined as the reversible work per unit area required to separate the two-phase interface into two free surfaces [23]. Therefore, *W_sep_* can be used to evaluate the interfacial bonding strength. When *W_sep_* is positive, it means that the two surfaces can form a stable interface, and the larger the value of *W_sep_*, the stronger the interatomic bonding force and the higher the interfacial bonding strength, while when *W_sep_* is negative, it means that the interface cannot exist stably. The equation for *W_sep_* of Ag/Ni is:

*W_sep_ = (E_Ag_ + E_Ni_ − E_Ag/Ni_)/S* (1)

where *E_Ag_* is the energy of the Ag surface in the Ag/Ni interface model, *E_Ni_* is the energy of the Ni surface in the Ag/Ni interface model, *E_Ag/Ni_* is the total energy of the Ag/Ni interface model, and *S* is the area of the interface.

The total energy, the lattice constants *a* and *b* required for the area, and *W_sep_* are presented in Table 2.

According to *W_sep_* in Table 2, it can be obtained that both the undoped and doped *W_sep_* are positive values, indicating that a stable interface can be formed. Moreover, the value after doping is larger, indicating that the bonding force between interface atoms is stronger after doping, and the interfacial bonding strength is higher. The order of *W_sep_* is: Ag/Ni-C > Ag/Ni-Cu > Ag/Ni, and the interfacial bonding strength after C doping is the highest, followed by Cu.

### 3.2. Interfacial Energy

The interfacial energy is the excess energy per unit area of the interface caused by the atomic distortion, chemical bond change, and structural strain at the interface when the interface is formed in the system [24]. Interfacial energy can often be used to evaluate interfacial stability. Since the interface structure mismatch can produce interface mismatch strain, the interface energy should be positive for interfaces composed of different solid-phase materials, and the smaller the positive value, the higher the interface stability. The formula for the interfacial energy (*γ_int_*) of Ag/Ni is:

(2) $$\gamma_{int}=\sigma_{Ag}+\sigma_{Ni}-W_{sep}$$

Among them, $\sigma =\frac{E_{{}_{slab}}^{}-\frac{N_{slab}}{N_{bulk}}E_{{}_{bulk}}^{}}{2A_{}}$, where *E_slab_* is the total energy of the optimized surface model, *E_bulk_* is the total energy of the optimized bulk unit cell model, *N_slab_* is the number of atoms contained in the surface model, *N_bulk_* is the number of atoms contained in the bulk unit model, and *A* is the area of the surface. Table 3 shows the interfacial energy of Ag/Ni and Cu, C-doped Ag/Ni.

According to the interfacial energy in Table 3, it can be obtained that the interfacial energy of undoped and doped was positive, indicating that there was good interfacial stability. The value of the interfacial energy of the doped interface models was greater than that of the Ag/Ni interface model, indicating that the stability of the doped models improved. The C-doped Ag/Ni demonstrated the strongest stability. Since the atomic radius and electronegativity of the dopant atoms are different from those of Ni atoms, the introduction of doped atoms will distort the lattice constant of the Ag/Ni interface and affect the stability.

### 3.3. Density of States

**Figure 2:**
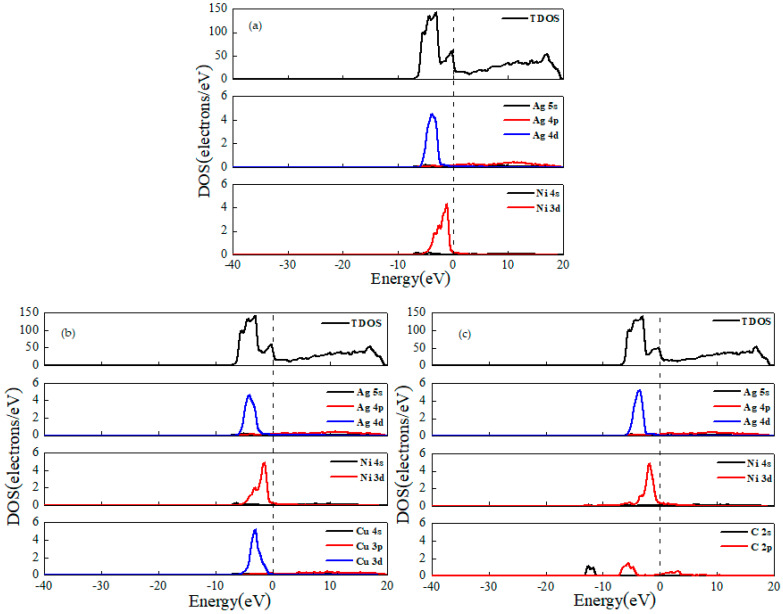
Density of states: (**a**) Ag/Ni, (**b**) Ag/Ni-Cu, and (**c**) Ag/Ni-C.

The density of states reflects the interaction of individual atoms and doped atoms with other atoms in the interface model, and provides the distribution pattern of electrons at different energy values. The density of states diagram is presented in Figure 2, where Figure 2a is the Ag/Ni, and Figure 2b,c are the Cu- and C-doped Ag/Ni, respectively. Total density of states (TDOS) represents the energy distribution state of all electrons in the system. The partial density of states indicates the bonding of different orbital electrons.

From the density of states of undoped Ag/Ni in Figure 2a, it can be seen that the number of electrons near the Fermi energy level was large, indicating that Ag/Ni has good conductivity. At −10~0 eV, the peaks of Ag/Ni mainly originate from the S orbital of Ag and the d orbital contribution of Ni, and in the region between −4 and −3 eV, there are obvious overlapping peaks of Ag-s and Ni-d orbital. Furthermore, in the region from 0 to 20 eV, the peak is mainly provided by the Ag-p orbital.

From the density of states of Cu-doped Ag/Ni in Figure 2b, it can be seen that at −10~0 eV, there is a Cu-d orbital in addition to the Ag-s orbital and Ni-d orbital contributions. There are obvious overlapping peaks of Ag-s orbitals between −5 and −1 eV, Ni-d orbitals, and Cu-d orbitals. Two-by-two hybridization is bonding orbitals. In the region from 0 to 20 eV, the peaks are mainly provided by the Ag-p orbital. From the density of states of C-doped Ag/Ni in Figure 2c, it can be seen that at −10~0 eV, there are C-p orbitals in addition to Ag-s and Ni-d orbitals’ contributions. C-p and C-d orbitals of Ni at −7~−4 eV and 0~3 eV, there is an electronic overlap for bonding orbitals. In the region from 0 to 20 eV, in addition to Ag-p orbitals, the peak also has C-p orbitals.

### 3.4. Mulliken Population Analysis

The charge population is used to characterize the transfer of electrons between atoms. For atoms that lose electrons, the charge population is positive. On the contrary, for atoms that are easy to gain electrons, the charge population is negative. The greater the absolute value of the charge population, the stronger the ability to gain or lose electrons. Table 4 shows the average charge population values of Ag/Ni and Cu, C-doped Ag/Ni interface models.

It can be seen from Table 4 that the Cu atom population was positive, and electrons were lost in the process of electron transfer. The C atom has a negative and larger charge population and a stronger ability to obtain electrons in the process of electron transfer. The ability of each atom to gain and lose electrons is redistributed after doping. The ability of Ag and Ni atoms to gain and lose electrons weakened after Cu doping, while the ability of Ni atoms to lose electrons enhanced after C doping.

Bond population is used to characterize the nature of bonding between atoms. The larger the absolute value of bond population is, the more electron clouds overlap each other, the stronger the bonding ability, and the more stable the chemical bond formed. The bond population values close to 0 are likely to be bonded and strongly ionic, whereas positive deviation from 0 is likely to be more covalent, the more covalent the bond may be, and the negative deviation from 0 is usually regarded as the existence of anti-bonding [25]. Table 5 presents the bond populations and average values for the various types of bonds corresponding to the Ag/Ni and Cu, C-doped Ag/Ni interface models.

It can be seen from Table 5 that the bond populations corresponding to the Ag-Ni bonds in the Ag/Ni interface model were all greater than 0 and had a certain covalency.

Ag-Ni at room temperature is an almost incompatible binary system. The calculation of the interface model revealed that the formation of covalent bonds between Ag and Ni atoms at the Ag/Ni phase interface was possible at the electronic structure level, which can contribute to the stability of the Ag/Ni interface. This is because of the large difference between the phase interface structure and the crystal structure of Ag or Ni, and there were more unsaturated bonds in the two-phase region. Therefore, it is possible to form Ag-Ni bonds in some phase interface regions of the Ag/Ni system from the atomic scale.

After Cu and C doping, the bond population between Ag and Ag was all positive and the average value increased, while the average bond population between Ni and Ni decreased, indicating that the formation of anti-bonding between Ni and Ni increased after doping, and the formation of bonding between Ag and Ag increased. After Cu doping, there was a certain bond population between Ag and Ni atoms and Ag and Cu atoms in the interface region. After C doping, there was a certain bond population between Ag and Ni atoms in the interface region. This is consistent with the interfacial bonding strength and density of states analysis. After Cu doping, Cu and Ni both had bonding and anti-bonding states, showing an overall anti-bonding state. This leaves the total energy of the system at a high level and reduces stability. After Cu and C doping, the maximum value of the bond population was larger than that of Ag-Ni, especially after C doping. Moreover, after C doping, more Ni atoms and Ag atoms formed covalent bonds. This also explains why the interfacial bonding strength and interfacial stability of C were better than those after Cu doping.

## 4. Experiment

### 4.1. Preparation of Ag/Ni Contact Materials by Powder Metallurgy

Ag/Ni and Cu, C-doped Ag/Ni were prepared by the high-energy ball mill with the powder metallurgy method. Firstly, powder metallurgy was used to mix powder on a high-energy ball mill so that the Cu- and C-doped Ni powder and Ag powder could be mixed uniformly in a vacuum ball mill. Then, primary pressing, primary firing (in a vacuum sintering furnace), re-pressing, re-firing (in a vacuum sintering furnace), and polishing were carried out to prepare Ag/Ni and doped Ag/Ni contact materials, followed by cutting to obtain contact samples of 3.2 mm.

The sample of Ag/Ni and Cu, C-doped Ag/Ni contacts prepared for this experiment was 10 g. The mass ratios of Ag, Ni, and the additives Cu and C required for the experiment have been presented in Table 1.

### 4.2. X-ray Diffraction Experiment

**Figure 3:**
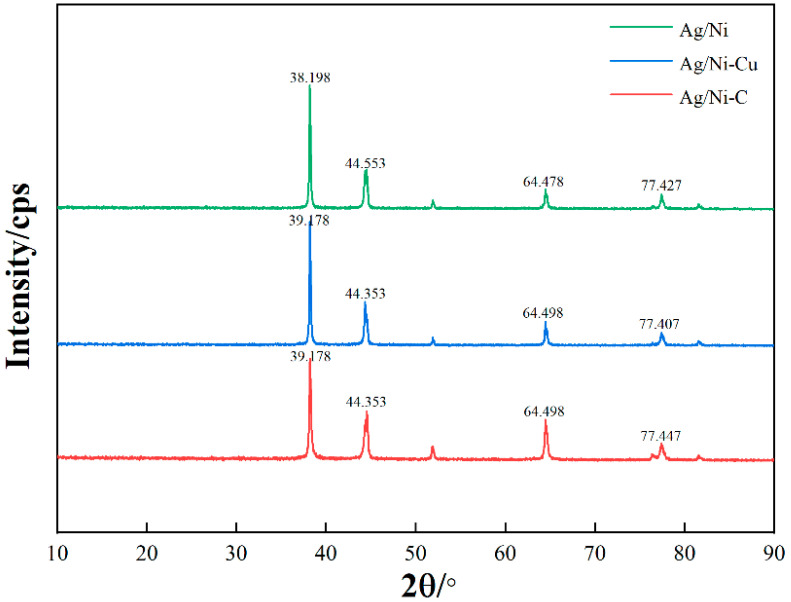
X-ray diffraction pattern.

To ensure that the experiment corresponds to the simulation model, a Bruker D8 DISCOVER model X-ray diffractometer (XRD) was used to analyze the phase structure of the prepared materials. The X-ray generator was Cu target Kα rays, the wavelength was 0.15405 nm, the scanning angle range was set to 10°~90°, and the scanning speed was 6°/min. The X-ray diffraction patterns of Ag/Ni and Cu, C-doped Ag/Ni samples are shown in Figure 3.

As can be seen from Figure 3, the XRD patterns of Cu- and C-doped Ag/Ni showed no diffraction peaks associated with the doped elements, which indicates that the doping did not change the crystal structure of Ni. Cu and C formed a solid solution with Ni, which corresponds to the substitution doping model established by simulation. The comparison shows that the positions of the characteristic diffraction peaks were basically the same for both, except for a slight shift in the 2θ angle of the characteristic diffraction peaks after doping, which was mainly due to the change in lattice constant caused by the doping element. The theoretical simulation model constructed in this paper matches the experiment.

### 4.3. Arc Energy Experiment

A JF04D electrical contact test system was used for the electrical contact experiment. The power-on protection of the test system was set to ±40 V, the experimental current was DC 15 A, the voltage was DC 24 V, the closing frequency between contacts was 60 times/min, the contact pressure was set to 86 cN, and the number of actions was set to 100,000 times.

**Figure 4:**
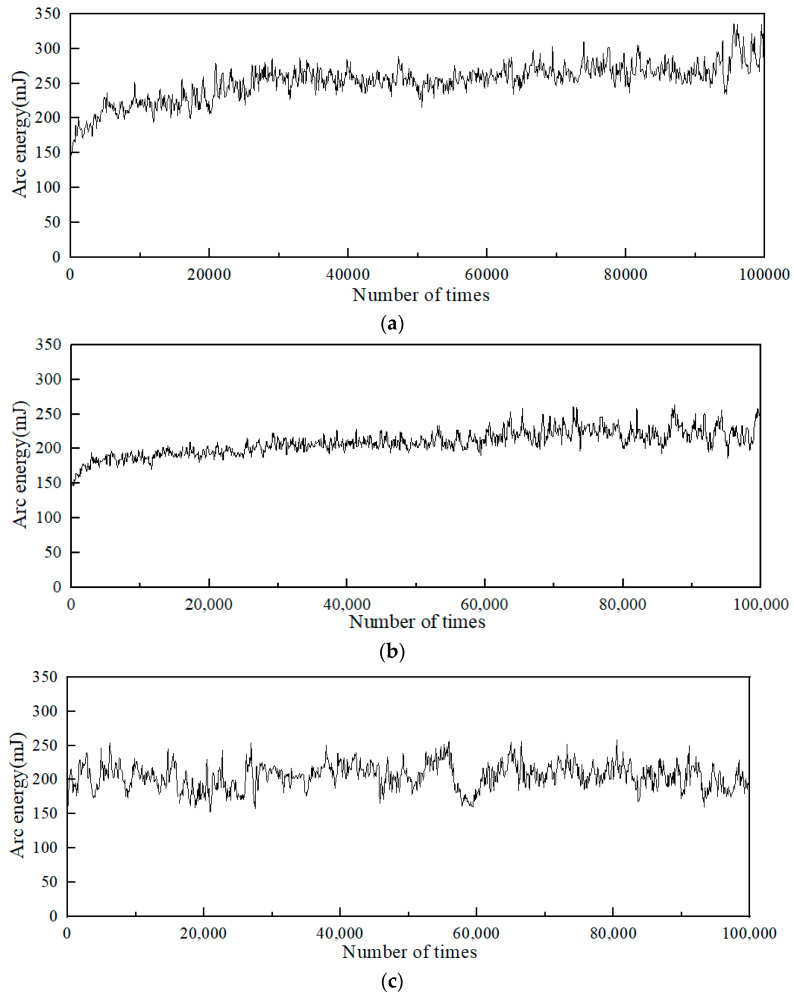
Arc energy: (**a**) Ag/Ni, (**b**) Ag/Ni-Cu, and (**c**) Ag/Ni-C.

During the opening and breaking process of the electrical contact, an arc will be generated due to the voltage and current, and the formation of the arc will release a large amount of energy. Electrical contact material in the heat input by the arc and the arc force will occur under the action of material evaporation, spattering, and other arc erosion. The variation curves of arc energy in the electrical contact experiments are presented in Figure 4. Among them, Figure 4a is the arc energy of Ag/Ni, and Figure 4b,c are the arc energies of Cu- and C-doped Ag/Ni, respectively.

It can be seen from Figure 4a that the arc energy curve of the Ag/Ni contact material showed an almost increasing trend, with the arc energy increased from 150 to around 340 mJ. Figure 4b,c show the arc energy curves of Cu- and C-doped Ag/Ni. Compared with the undoped Ag/Ni contact material, the addition of doping elements significantly reduced the arc energy of Ag/Ni. The arc energy curve of Ag/Ni after Cu doping was relatively stable, but the arc energy trend of Cu was larger than that of C-doped Ag/Ni. The arc energies of Ag/Ni and Cu-doped Ag/Ni both showed an upward trend. However, the arc energy did not show an upward trend after C doping.

### 4.4. Welding Force Experiment

The contact fusion welding is closely related to the experimental current, voltage, contact pressure, contact material, and other factors. The welding force obtained from the electrical contact experiment can be used to evaluate the welding resistance of the electrical contact. The smaller the fusion welding force, the better the welding resistance of the material.

**Figure 5:**
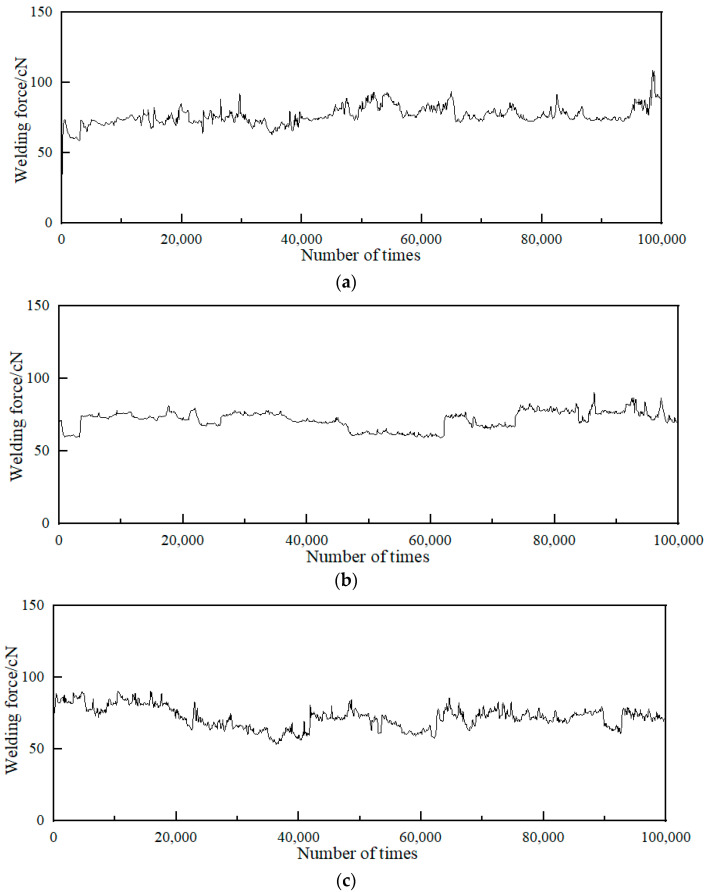
Welding force: (**a**) Ag/Ni, (**b**) Ag/Ni-Cu, and (**c**) Ag/Ni-C.

Figure 5 shows the change curve of the welding force in the electrical contact experiment. Among them, Figure 5a is the welding force of Ag/Ni, and Figure 5b,c are the welding force of Cu- and C-doped Ag/Ni, respectively.

From the welding force curve of undoped Ag/Ni in Figure 5a, it can be seen that the welding force of undoped Ag/Ni fluctuated considerably on the whole. There was an abrupt change at the 20,000th and 25,000th times, where the welding force dropped sharply from 75 to 45 cN, then rose to 85 cN at the 30,000th time, and then dropped to about 70 cN. Then it increased, and there was still a large degree of abrupt change at the 50,000th, 65,000th, and 98,000th times. The welding force of undoped Ag/Ni fluctuated obviously, and the stability was poor. Figure 5b,c are the welding force curves of Cu- and C-doped Ag/Ni. Compared with undoped Ag/Ni, the welding force decreased, and the curve after Cu doping became relatively flatter with less abrupt changes and more stable overall. After C-doping Ag/Ni, the welding force was relatively large in the early stage of the experiment, and it was unstable compared with Cu doping. In the later stage of the experiment, C doping showed a better result than Cu doping, which was consistent with the trend of arc energy. The doping elements can reduce the welding force of Ag/Ni and improve the welding resistance of Ag/Ni contacts.

To compare and analyze the influence of doping on the welding resistance of Ag/Ni contact materials, the minimum, maximum, and average values of arc energy and welding force of Ag/Ni and Cu, C-doped Ag/Ni are shown in Table 6.

It can be seen from Table 6 that the average arc energy, average welding force, maximum arc energy, and maximum welding force of the Ag/Ni contact material doped with Cu and C were reduced, indicating that doping can improve the welding resistance of Ag/Ni contact materials. The activation effect of doping elements can effectively increase the solubility of Ni in the Ag liquid phase and improve the interfacial bonding ability of Ni and Ag, which in turn improves the welding resistance of Ag/Ni contact materials. The fusion welding resistance of Ag/Ni material is mainly related to the fine particles NiO, Ni, and pores in its surface layer. C has good physical and chemical properties, such as a high melting point and reducibility, as well as its honeycomb structure. Under the action of an arc, carbon or carbon monoxide can be used to inhibit the oxidation of Ni and strengthen the reduction of NiO, which is conducive to increasing the content of Ni in the surface layer, inhibiting the formation of a strong connection between Ag and Ag in the material after arc ignition, controlling the arc in the local area, where it triggers ignition and improves the fusion welding strength. As a result, C doping showed better welding resistance than Cu doping in the later stage of the experiment.

## 5. Conclusions

In this paper, the first-principles method was used to calculate the interface model of Cu, C-doped Ag/Ni to obtain the interfacial bonding strength, interfacial energy, density of states, and population. Finally, Cu, C-doped Ag/Ni contact materials were prepared by high-energy ball milling and the powder metallurgy method. The results showed the following:

(1) The simulation analysis showed that the interfacial bonding strength and stability of the doped material improved, and the best interfacial bonding strength and stability were seen for the interface model of C-doped Ag/Ni. The analysis of electronic structure revealed that there are covalent bonds between Ag and Ni atoms of the Ag/Ni phase interface at the electronic structure level. After doping, the bond population between Ni and Ag and the interfacial bonding strength increased. Compared with Cu doping, more Ni atoms formed covalent bonds with Ag atoms. Cu has more anti-bonding states than C doping, so the interfacial stability was not as good as C doping.
(2) Through experimental analysis, it was found that the average arc energy and welding force of Ag/Ni contact materials were reduced after doping, and the average arc energy and welding force of C element doping were lower. C doping showed better welding resistance than Cu element doping, especially at the later stage of the experiment. Therefore, the C-doped Ag/Ni contact material had the best welding resistance.

Overall, the simulation results showed that the interface performance of the contact material doped with C was the best. Additionally, the simulation result was verified through experiments, providing an effective method for the research of Ag/Ni contact material.

## Figures and Tables

**Table 1:** Atomic ratio and mass ratio.

Material	Atomic Ratio	Mass Ratio
Ag:Ni	36:12	84.65%:15.35%
Ag:Ni:Cu	36:11:1	84.56%:14.06%:1.38%
Ag:Ni:C	36:11:1	85.52%:14.22%:0.26%

**Table 2:** The work of interfacial separation.

Model	Energy (eV)	A (Å)	B (Å)	*W_sep_* (eV/Å^2^)
Ag/Ni	Ag: −144,212.05	12.493	2.8887	0.0352
	Ni: −16,341.01			
	AgNi15: −160,554.33			
Ag/Ni-Cu	Ag: −144,212.05	12.379	2.8926	0.0379
	NiCu: −16,659.89			
	AgNi15Cu: 160,873.30			
Ag/Ni-C	Ag: −144,212.05	12.235	2.9020	0.0411
	NiC: −15,135.99			
	AgNi15C: −159,349.50			

**Table 3:** Interfacial energy.

Model	*σ_Ag_* (eV)	*σ_Ni-X_* (eV)	*γ_int_* (eV/Å^2^)
Ag/Ni	0.0384	0.1495	0.1527
Ag/Ni-Cu	0.0384	0.1439	0.1444
Ag/Ni-C	0.0384	0.1021	0.0993

**Table 4:** Mulliken charge population.

Model	Atom Population			
	Ag	Ni	Cu	C
Ag/Ni	−0.00417	0.0142		
Ag/Ni-Cu	−0.00333	0.00273	0.05	
Ag/Ni-C	−0.00306	0.073		−0.69

**Table 5:** Bond populations and average values for various types of bonds.

Model	Bond Populations/Average Values				
	Ag-Ag	Ag-Ni	Ni-Ni	Ag-X	Ni-X
Ag/Ni	−0.04~0.39/0.249	0.12~0.41/0.268	−0.38~0.63/0.311		
Ag/Ni-Cu	0.01~0.38/0.254	0.42/0.420	−0.42~0.63/0.135	0.11/0.110	−0.56~0.34/−0.028
Ag/Ni-C	0.07~0.38/0.312	0.15~0.49/0.340	−0.36~0.58/0.133		0.17~0.79/0.493

**Table 6:** Arc energy and welding force.

Material	Arc Energy/mJ			Welding Force/cN		
	Minimum	Maximum	Average	Minimum	Maximum	Average
Ag/Ni	146.4	336.8	252.5	58.5	108.7	76.4
Ag/Ni-Cu	146.0	263.9	209.1	59.5	90.5	73.5
Ag/Ni-C	151.8	258.7	204.9	52.8	90.4	71.9

## Data Availability

The data of this study are available from the corresponding author upon reasonable request.
